# Diet and Nutrition in Cancer Survivorship and Palliative Care

**DOI:** 10.1155/2013/917647

**Published:** 2013-10-30

**Authors:** Anthony J. Bazzan, Andrew B. Newberg, William C. Cho, Daniel A. Monti

**Affiliations:** ^1^Myrna Brind Center of Integrative Medicine, Thomas Jefferson University, Philadelphia, PA 19107, USA; ^2^Department of Clinical Oncology, Queen Elizabeth Hospital, Kowloon, Hong Kong

## Abstract

The primary goal of palliative cancer care is typically to relieve suffering and improve quality of life. Most approaches to diet in this setting have focused only on eating as many calories as possible to avoid cachexia. However, as the concept of palliative care has evolved to include all aspects of cancer survivorship and not just end of life care, there is an increasing need to thoughtfully consider diet and nutrition approaches that can impact not only quality of life but overall health outcomes and perhaps even positively affect cancer recurrence and progression. In this regard, there has been a recent emphasis in the literature on nutrition and cancer as an important factor in both quality of life and in the pathophysiology of cancer. Hence, the primary purpose of this paper is to review the current data on diet and nutrition as it pertains to a wide range of cancer patients in the palliative care setting.

## 1. Introduction

In cancer care models, the primary goals of palliative care are to relieve suffering and improve quality of life across the cancer illness trajectory. As the concept of palliative care has evolved to include all aspects of cancer survivorship and not just end of life care, there is an increasing need to thoughtfully consider lifestyle behaviors that can positively affect health outcomes and cancer progression for those who have responded to oncological interventions. In that regard, there has been a recent emphasis in the literature on nutrition and cancer as an important factor in both quality of life and in the pathophysiology of cancer. Hence, the primary purpose of this paper is to review the current data on diet and nutrition as it pertains to a wide range of patients with cancer in the palliative care setting. 

 At one time, palliative care referred to “end of life” only, in which case patients were encouraged to eat whatever they felt like. If the patient deteriorated, the standard nutritional strategy became more focused on quality of life (QOL) and relief of symptoms, rather than active nutritional interventions aimed at improving outcome. At this stage of the cancer illness, the expectation was short survival time, and the major goal was to minimize cachexia. Thus, any calories were deemed to be “good” calories. As a broader number and range of patients come under the purview of this expanded palliative model, recent research has suggested that it may be more appropriate to develop a specific nutritional strategy which can help improve the overall health and well-being of cancer patients, including those in the traditionally defined palliative care setting. In fact, when making decisions about nutritional support, one must consider diagnosis, prognosis, current status and symptoms, pathways of disease progression, treatment and possible side effects, patient comfort and peer and social support, socioeconomic status, cultural and religious views, and ethical and legal issues. 

 Hence, the primary purpose of this paper is to introduce the concept of diet as a potential survivorship intervention as opposed to sustenance only. Our goal is to synthesize the known knowledge in a way that might assist clinicians while making nutritional recommendations to

1. ensuring an adequate amount of calories,
2. reducing foods and dietary habits that have been specifically linked to modifiable risk factors for cancer reoccurrence,
3. creating a diet that minimizes inflammation, insulin resistance, and oxidative stress,
4. ensuring an adequate amount of specific nutrients and selective nutritional supplementation linked to cancer prevention/recurrence.

Using these strategies and based upon the most current data available, appropriate, individualized approaches to palliative care diet and nutrition can be established for a wide spectrum of patients with cancer. The diagnosis of cancer often powerfully motivates survivors to alter their lifestyle habits, so healthcare providers need to be aware of what changes patients are likely and willing to make in order to determine the most appropriate recommendations [1]. This review will explore the relevant data and make recommendations for dietary and nutritional considerations in the palliative care setting. 

It should be stated at the outset that this paper pertains broadly to all types of cancer. However, we also recognize that there may be differences in the nutritional approach to patients with different types of cancers. Thus, patients with lymphoma may have different requirements or benefit differently from various supplements than patients with sarcoma. At the present moment, we do not feel that there is sufficient data to help with these distinctions. In addition, this paper pertains primarily to adult patients rather than children. While some of the concepts below would certainly pertain to pediatric patients and an overall analysis of nutrition in children would be extremely valuable, space does not permit an additional review and analysis of this area of palliative care.

## 2. Ensuring an Adequate Amount of Calories

Nutrition should be a priority early in palliative care. Food has a primary role in life for everyone including people with advanced illness. Adequate nutrition is essential, not only to meet physiological requirements, but also because it has additional psychological, spiritual, social, and cultural benefits for patients and caregivers. Strategically addressing these additional aspects is of high importance in the palliative setting. 

 The hypothesis that increased energy expenditure leads to weight loss experienced by patients with cancer has been supported in the literature [2, 3]. However, other studies showed that the effect of cancer on energy expenditure is variable and complex [4]. Thus, patients with cancer can be in a physiologically hypermetabolic, hypometabolic, or eumetabolic state. The particular state of an individual patient is not fully explained by differences in tumor type, tumor burden, nutritional status, or duration of disease. However, tumor type has been found to be more predictive. For instance, gastric, pancreatic, and biliary cancers are typically more hypermetabolic than nongastrointestinal cancers. The lack of a clear relationship between clinical status and metabolic status makes it hard to generalize caloric recommendations. Therefore, recommendations for nutrition and physical activity for persons who are living with advanced cancer are likely best made based on their individual needs and clinical issues.

The American Cancer Society (ACS) provides some important guidelines for nutritional support in patients with cancer. The ACS guidelines acknowledge that “many persons with advanced cancer may need to adapt food choices and eating patterns to meet nutritional needs and to manage symptoms and adverse effects such as pain, constipation, and loss of appetite” [5]. 

 The specific dietary guidelines from the ACS, however, are relatively general and include choosing foods and drinks in amounts that help patients get to and maintain a healthy weight; limiting the amount of processed and red meat eaten; eating at least 2(1/2) cups of vegetables and fruits each day; and choosing whole grains instead of refined grain products. These guidelines mirror others that emphasize high-calorie, high-energy meals in small portions [6], and such an approach may not only satisfy caloric requirements but also positively impact secondary problems related to sugar consumption, insulin resistance, inflammation, and others. Diet may also have a role in alleviating treatment-related symptoms. For example, opiates to relieve pain commonly cause constipation, but this side effect may be lessened by a diet high in fluid and fiber. In addition, some medications are included in the ACS guidelines which cite the use of nonsteroidal anti-inflammatory drugs or omega-3 fatty acid supplements that may help stabilize or improve body weight, along with nutritional and functional status [7–9].

 The notion of palliative care traditionally evoked a cachectic picture. However, many patients are overweight at the time of diagnosis or become so after treatment [10]. Women diagnosed with early-stage breast cancer might improve overall prognosis and survival by adopting more healthful dietary patterns [11]. Health care professionals should encourage weight management at all phases of the cancer care continuum as a means to potentially avoid adverse sequelae, as well as to improve overall health and possibly survival [12]. Comprehensive approaches that involve dietary and behavior modification and increased aerobic and strength training exercise have shown promise in either preventing weight (fat) gain or promoting weight (fat) loss, reducing biomarkers associated with inflammation and comorbidity and improving lifestyle behaviors, functional status, and QOL in this high-risk patient population [13].

 Hence, optimal body mass index and body metrics should be pursued in an appropriate manner based upon the status of the patient, whether cachectic or overweight. The health care team should be intimately involved in addressing the specific caloric needs of each cancer patient throughout the clinical course, both in terms of the number of calories required and the quality of those calories.

## 3. Reducing Foods and Dietary Habits Specifically Linked to Cancer Recurrence

Eating foods that contain high amounts of excess sugars is an unhealthy diet even in the palliative care setting. Such energy-dense, nutrient-poor foods include refined sugars, candies, fried foods, and so-called “junk food.” The literature contains ample consensus that eating such foods favors the onset of obesity and diabetes, both factors in cancer development. For example, a high intake of refined carbohydrates has been associated with cancer in general [14] and more specifically with prostate cancer [15]. Fructose intake, which is also associated with obesity, has been shown to be used preferentially by pancreatic cancer cells to grow [16] and cancer of the small intestine [17]. Diabetes mellitus also has been linked to cancer [18, 19].

 Red and processed meat consumption has consistently gained a reputation as a contributor to disease, including cancer. Data is emerging that red and processed meats may influence disease recurrence and mortality as well, for example, for colorectal cancer survivors. Evidence shows that consumption of red meat can activate cancer genes in the colon [20] such as the MDM2 and ubiquitin genes as well as the WNT gene signaling pathway which is involved in epithelial proliferation and differentiation. Such genetic modulation can facilitate cellular progression to colon cancer [21, 22]. Prospective observational studies have shown that increased exercise after diagnosis and avoidance of a Western pattern diet (high intake of red and processed meats and refined grains) are associated with a reduced risk of cancer recurrence and improved overall survival in early-stage colorectal cancer after standard therapy [23].

 The data also suggests that red meat, in particular, is pro-inflammatory and procarcinogenic. For example, the European Prospective Investigation into Cancer and Nutrition-Potsdam study of 2,198 men and women found that the consumption of red meat was significantly associated with higher levels of the inflammatory markers GGT and hs-CRP when adjusted for potential confounding factors related to lifestyle and diet [24]. Another study showed that when people were given a 7-day dietary red meat intervention, fecal water genotoxicity significantly increased in response to the red meat intake [20]. The authors reported that genes significantly correlating with the increase in fecal water genotoxicity were involved in biological pathways indicative of genotoxic effects. These effects included modifications in DNA damage repair, the cell cycle, and apoptosis pathways. Thus, red meat should be minimized or eliminated from the diet of palliative care patients. However, it is important to ensure that patients continue to receive nutrients commonly found in meats such as iron, vitamin B, and essential amino acids. Thus, care should be taken to provide supplements when necessary to augment these requirements.

 A mouse study demonstrated that a high animal fat diet could actually induce tumor growth and progression, including epithelial-mesenchymal transition (EMT) and inflammation [21]. This study also showed the molecular mechanisms. Changes occurred through activation of mitogen-activated protein kinase and phosphatidylinositol-3-kinase signaling pathways. Importantly, the authors noted that a high animal fat diet could suppress p21(CIP1/WAF1) expression through increases in the nuclear histone deacetylase complex which leads to the activation of oncogenic reactions that involve EMT and inflammation in colorectal cancer.

 A dietary analysis of 1171 patients with bladder cancer compared a variety of dietary factors to those of 1,418 non-cancer patients [25]. The results showed that processed meat intake was positively associated with bladder cancer (with a significant odds ratio (OR) of 1.28 between the highest versus lowest quartile of meat intake, *P* < 0.05), with a stronger association for processed red meat. In this study, the intake of fruits or vegetables had no correlation with bladder cancer, but higher levels of vitamin B12 intake were found to be protective (individuals in the highest, compared with the lowest, quartile of vitamin B12 intake had a lower risk of bladder cancer with an OR of 0.77, *P* < 0.05). 

 In an analysis of patients with early stage breast cancer from the Life After Cancer Epidemiology Study [11] researchers compared 1,901 patients diagnosed with early stage breast cancer. Two dietary patterns were identified: prudent (high intakes of fruits, vegetables, whole grains, and poultry) and Western (high intakes of red and processed meats and refined grains). The results showed that increased adherence to a prudent dietary pattern was associated with a statistically significant decreased risk of overall death and also death from nonbreast cancer causes. The authors reported that increasing consumption of a Western dietary pattern was related to an increased risk of overall death and also death from nonbreast cancer causes. Interestingly, neither dietary pattern was associated with the risk of breast cancer recurrence or death from breast cancer [11]. Further, the observations were generally not modified by physical activity, being overweight, or smoking. Thus, more research is needed to elucidate these issues in the broader context of patients with cancer.

 Overall, the cumulative data suggests that refined carbohydrate and red meat consumption should be avoided or significantly reduced in the diet of patients with cancer as there are many other healthier sources of caloric intake. Achievement and maintenance of a healthy body composition via a plant-based diet high in fruits, vegetables, and whole grains and low in saturated fats and red or processed meats should be the guidelines imparted to patients from their health care providers. 

## 4. Diets That Minimize Inflammation and Oxidative Stress

As it has become increasingly apparent that cancers use inflammatory pathways for growth and metastases, the concept of utilizing anti-inflammatory diets has received more attention. The literature indeed shows more studies exploring the link between cancer and inflammation [26]. Inflammation itself is associated with high levels of oxidative stress that can damage most of the body's tissues and genetic material which ultimately can lead to cancer formation [27]. In integrative medicine, there is a long tradition of utilizing anti-inflammatory diets to attenuate the negative effects of inflammation and oxidative stress. Ancient cultures also developed and used anti-inflammatory diets, such as in Ayurvedic (a system of traditional medicine native to the Indian subcontinent that stresses plant based treatment), which are now investigated using modern criteria [28].

 Mounting evidence suggests that chronic inflammation mediates most chronic diseases, including cancer, and causes a great deal of morbidity and ultimately mortality. Hence, good targets for anticancer strategies include major regulators of inflammation, cellular transformation, tumor cell survival, proliferation, invasion, angiogenesis, and metastasis such as transcription factors nuclear factor-kappa B (NF-*κ*B), STAT3, and eIF4E activity [29]. Agents that can inhibit NF-*κ*B and STAT3 activation pathways have the potential to prevent and/or treat cancer and to be of use in the palliative care setting. For instance, compounds called triterpenes are particularly adept at suppressing inflammatory pathways linked to cancer. The mechanism of action is most likely related to the suppression of different cytokines, in addition to prostaglandin E_2_, particularly from activated macrophages [30]. These triterpenes include avicins, betulinic acid, boswellic acid, celastrol, diosgenin, madecassic acid, maslinic acid, momordin, saikosaponins, *Platycodon*, pristimerin, ursolic acid, and withanolide. These can be obtained from botanical medicines and diet [31]. 

It is known that a major risk factor for sporadic colon cancer is a high animal fat Western diet, which has been linked to a cancer-prone, proinflammatory state [32]. Diets with an emphasis on fresh fruits and vegetables are associated with lower cancer incidence [33]. Diet has the potential to encourage colon cancer development, but on the other hand, recent evidence demonstrates that certain natural dietary products such as beans [34] and apples [35] can halt colon cancer development and progression via epigenetic regulation. Another study suggested that unfavorable diet-promoted epigenetic dysregulation may contribute to inflammation-driven diseases, such as cancer, via inappropriate silencing of genes necessary to inhibit cancer development [36]. 

 An interesting animal study explored whether compound K, a metabolite of American ginseng, could help reduce inflammation and also the risk of cancer in mice with xenograft colon cancer [37]. The results showed that ginseng significantly inhibited colonic inflammation and tumor growth. Ginseng could reduce proliferation by 50% and increased apoptosis by 50% in colon tumor cells. The epidermal growth factor receptor (EGFR) cascade was upregulated in colon cancer, but administration of ginseng significantly reduced EGFR activation. In addition, the authors reported that dietary ginseng altered colonic microbial diversity and that compound K significantly inhibited tumor xenograft growth. Some of the more readily available supplemental agents that can be taken both as part of food and as commercial products include the following.

Ginger, curcumin/turmeric, *Boswellia serrata*, and American ginseng. These compounds have both anti-inflammatory and antioxidant activity.

The relationships between energy intake, energy density, and energy balance are affected by systemic inflammation. Thus, targeting systemic inflammation is likely to be important in nutritional interventions in palliative care patients. Wholesome diets rich in fresh and cooked vegetables, lean protein, coupled with the use of probiotics, prebiotics, omega-3 polyunsaturated fatty acids, and vitamin D_3_, are a usual part of the recommendation from our Integrative Medicine Center [38]. This combination of foods and appropriate supplements can have a profound anti-inflammatory effect in the gut and body [39–41]. 

## 5. Specific Nutrients and Selective Nutritional Supplementation Linked to Cancer Prevention/Recurrence

As many as 50% or more of patients with cancer take vitamins, herbal preparations, and other supplements without medical guidance [42, 43]. On one hand, this is important information for the clinician who should be aware of the perils of some supplements such as herb-drug interactions (e.g., St. John's wort and irinotecan). If physicians are not aware of any potential interactions, there are many resources (specifically the medical literature) to provide guidance regarding the use of different nutritional supplements in patients with cancer. In addition, physicians should consider the judicious use of specific nutritional supplements that might be beneficial in the cancer or palliative care setting. In fact, there is a growing research base upon which to develop a specific approach to utilizing nutritional supplements in the palliative care setting. It should be noted that taking supplements must be weighed against other medications that the patient may be on to ensure that there are no potentially adverse interactions. However, most supplements are safe and can be used in combination with medications with the help of experienced practitioners. Data support the use of supplements to provide nutritional support not obtained with the patient's current diet and with the intent to ameliorate the specific pathophysiological status and needs of the patient. Supplements can be considered to support the proper immune function, and balanced endocrine function. With these specific goals in mind, the research data supports a potential core supplement program that can be introduced into patient care. Such a program should utilize probiotics, prebiotics, omega-3 fatty acids, and vitamin D since each of these provides important health benefits in the palliative care and cancer setting. It should also be noted that good diets do not automatically supplement these aspects of nutrition adequately, and therefore, such supplements should be considered in virtually every patient. As above, correcting these unmet needs addressing immune function, inflammation, and micronutrient status may deliver health benefits and possibly have the patient experience less complications from the disease and the treatments. 

### 5.1. Probiotics and Prebiotics

Use of probiotics and prebiotics is gaining support in view of recent studies. Probiotics are microbiota whose biology is overall symbiotic and favorable to health in humans [44]. Probiotics may play an important role as the body contains at least tenfold more bacterial cells than human cells, mostly in the intestinal tract. Intestinal health is critically supported by this internal microbial community. Symbiosis in this biomass is crucial for maintaining a healthy balance within the host-diet-microbiota triangle. Detrimental changes in any of these three factors may drive a healthy situation into a state of disease, and disease states are often associated with an imbalance in this triangle. The role of probiotics in the cancer field is just recently starting to attract attention. This is happening from such areas as probiotic diet-based prevention and even includes the treatment of cancer. One paper states that “Researchers are in universal agreement with the critical role of probiotics in getting rid of mutagens, delaying the onset of tumors, alleviating the side effects, pepping up chemotherapy, easing the postoperative complications, foiling remission and lifting the spirit of survivors” [45]. “Microbiota influence the health of the host, and the science of host-microbe mutualism is a rapidly expanding field. Both luminal diseases and systemic diseases (e.g., diabetes mellitus) can be influenced by the microbiota. It seems very plausible that one mechanism by which our diet influences colorectal cancer is through changes in the microbiota” [46]. 

 Evidence supports a relationship between colorectal cancer and prebiotic, probiotic, symbiotic, and dysbiotic bacteria [47, 48]. Many practitioners and patients may not be aware of the existence and role of “prebiotics.” Prebiotics are fermentable ingredients that specifically target components of the indigenous microbiota known to be beneficial. They are short-chain low-digestible carbohydrates (LDCs) metabolized by gut microbiota and are used as an energy source, immune system enhancers, or facilitators of mineral uptake. Prebiotics include inulin (a chicory fructan) and fructo-oligosaccharides, which are natural to artichokes, asparagus, bananas, chicory, garlic, leeks, oats, onions, soybeans, and wheat. They stimulate the growth and activity of intestinal microorganisms that promote the health and well-being of the host. Intake of foods containing LDCs can improve the state of health and may prevent diseases such as certain forms of cancer [49]. The combination of pro- and prebiotics has excellent potential to effectively help the restoration of immune system function to more physiological states. Other oligosaccharides, such as xylose, maltose, and mannose, also may act as prebiotics [50]. Lactulose is perhaps one of the prebiotics most familiar to physicians.

 Regarding probiotics, the gut immune system is constantly exposed to multitudes of antigens contained in the environment and food. Peyer's patches and lymphoid follicles respond to antigenic stimuli releasing cytokines or producing antibodies (e.g., secretory IgA). Symbiotic intestinal microbiota generate responses that help reduce inflammation and enhance the health of the gut. Dysbiotic segmented filamentous bacteria induce Th17 cells and promote inflammation, which is a factor in cancer development. Unfavorable microbiota and their toxic components have been shown to act on both Nod1 and Nod2 receptors, and their defective signaling may account for the development of inflammatory bowel disease (IBD). IBD increases the risk of colorectal cancer [51]. 

 Beneficial intestinal microbiota due to their regulatory function of gut immune response can prevent or retard neoplastic growth. Probiotic bacteria can produce metabolites such as conjugated linoleic acid (CLA), a polyunsaturated fatty acid that has anticarcinogenic effects. In one study, mice treated with CLA or a probiotic called VSL#3 (contains 450 billion bacteria including *Streptococcus thermophilus, Bifidobacterium breve, Bifidobacterium longum, Bifidobacterium infantis, Lactobacillus acidophilus, Lactobacillus plantarum, Lactobacillus paracasei, and Lactobacillus delbrueckii spp. bulgaricus*) recovered faster from the acute inflammatory phase of IBD and had lower disease severity in the chronic phase. Adenoma and adenocarcinoma formation was also diminished by both treatments. VSL#3 increased the mRNA expression of TNF-*α*, angiostatin and PPAR*γ*, whereas CLA decreased COX-2 levels. Both CLA and VSL#3 suppressed colon carcinogenesis, although VSL#3 showed greater anti-carcinogenic and anti-inflammatory activities than CLA. CLA modulated expression of COX-2 levels in the colonic mucosa, whereas VSL#3 targeted regulatory mucosal CD4+ T cell responses [52]. Probiotics may suppress the growth of bacteria that convert procarcinogens into carcinogens, thereby reducing the amount of carcinogens in the intestine [41].

 Since cancers of the gastrointestinal tract may account for 25% of all cancers and for 9% of cancer deaths worldwide, we can see how the exogenous administration of synergistic bacterial strains (probiotics) has been more frequently suggested to influence various processes associated with an increased cancer risk. So far, mechanisms that could explain the preventive action of probiotics against colorectal cancer onset may include (a) binding and degradation of potential carcinogens; (b) quantitative, qualitative, and metabolic alterations of the intestinal microflora; (c) production of anti-tumorigenic or antimutagenic compounds; (d) competition with putrefactive and pathogenic microbiota; (e) enhancement of the host's immune response; (f) direct effects on cell proliferation; (g) improvement of the host's immune response; (h) antiproliferative effects via regulation of apoptosis and cell differentiation; (i) fermentation of undigested food; and (j) inhibition of tyrosine kinase signaling pathways [53, 54]. 

 Particularly meaningful to patients in treatment regimens that include radiation therapy (RT) to the abdominal region for cervical, ovarian, prostate, sigmoid, or colorectal cancer is the potential therapeutic benefit from probiotics. RT can upset the colonization resistance of the indigenous gut flora, causing RT-induced diarrhea, enteritis, and colitis sometimes in more than 80% of patients. Randomized trials have demonstrated efficacy of probiotics such as VSL#3 and *Lactobacillus casei* DN-114 001 in decreasing the incidence and grade of RT-induced diarrhea [55]. Probiotic lactic acid-producing bacteria are an easy, safe, and feasible approach to protect patients with cancer against the risk of radiation-induced diarrhea [56, 57]. Similarly, VSL#3 is effective for preventing severe diarrhea following chemotherapy with irinotecan and therefore could be a good choice for patients with cancer [58]. *Lactobacillus GG* supplementation may reduce the frequency of severe diarrhea and abdominal discomfort related to 5-FU-based chemotherapy [59]. 

 Symbiotics, the combination of probiotics and prebiotics, have been studied in patients with advanced colorectal cancer, who may have associated comorbidities such as reduction of immunity, increased rate of infections, impaired cicatrisation of wounds, and muscle weakness. The role of chronic inflammation is known in many of these complications. Investigators are in pursuit of knowledge in this field. The Human Microbiome Project holds promise to help us gain factual and useable knowledge to help patients with cancer [60]. This NIH funded study evaluated 300 patients (149 men and 151 women) with the goal of obtaining specimens from the oral cavity, nares, skin, gastrointestinal tract, and vagina in a longitudinal manner over approximately one year. The data from this study are still pending, but the overall approach focuses on the importance of the bacterial milieu of the patient as it relates to various disease. For example, evidence from this program suggests that the gut microbiota affect nutrient acquisition, energy harvesting, and a myriad of host metabolic pathways [61]. Existing data supports the use of probiotics in specific clinical scenarios as shown in the following. 

*Overview of Probiotics*. Probiotic type/benefits/dose.

- *L. acidophilus* and *B*. *delbrueckii* subspecies bulgaricus: antibiotic-associated diarrhea 2 × 10^9^ daily for 5–10 days.
- *L. acidophilus* and *B*. *longum*: antibiotic-associated diarrhea 5 × 10^9^ daily for 7 days.
- *L. rhamnosus GR-1* and *L. fermentum RC-14*: vulvovaginal candidiasis 10^9^ bacteria in skim milk, twice daily (taken orally) for 14 days.
- VSL#3 irritable bowel syndrome 9 × 10^11^ daily for 8 weeks.
- Ulcerative colitis active flares: 1.8 × 10^12^ twice daily for 6 weeks plus conventional therapy.
- Pouchitis 1.8 × 10^12^ bacteria twice daily.

It has been noted that “despite the positive results and plethora of agents, bacterial combinations and concentrations, the inconsistency in administration, the inhomogeneity of comparison groups and lack of stringent clinical endpoints remain obstacles in the effort to establish a definitive clinical strategy at this time. Further work is warranted to gain a keen understanding of their clinical value in CRC patients” [62]. It should be noted that while probiotics may be of help, in certain clinical settings, they might be not advisable. For example, probiotics are contraindicated in several inpatient populations [63, 64] including (1) patients with neutropenia or other causes of immunosuppression; (2) all intensive care unit patients; (3) all patients with central venous catheters receiving parenteral nutrition; and (4) all patients requiring administration of the probiotic via a feeding tube or requiring opening of capsules/crushing of medications for drug administration. 

 However, it is not yet possible to make generalized recommendations on when and when not to take specific prebiotics and probiotics at this time. Based on the available evidence, it is reasonable that physicians and patients discuss these issues together and come up with personal strategies using available products, sound clinical judgment, and the best current research evidence.

### 5.2. Omega-3 Fatty Acids

There is accumulating evidence that the use of omega-3 fatty acids has the potential as anticancer compounds. How they interact with the cancer process is becoming clearer as investigations unfold. “Diets rich in omega-3 polyunsaturated fatty acids (*ω*3-PUFAs) such as alpha-linolenic acid, eicosapentaenoic acid, and docosahexaenoic acid are associated with a decreased incidence and severity of several chronic diseases including cardiovascular disease (CVD) and cancer” [65]. As discussed earlier, it is known that inflammation contributes to tumor initiation, progression, and growth. Omega-3 fatty acids have known anti-inflammatory effects, and their role in cancer prevention and in cancer treatment is becoming better defined. *ω*3-PUFAs such as eicosapentaenoic acid (EPA) and polyphenols such as curcumin and resveratrol have been demonstrated to have anticolorectal cancer activity in preclinical models. The EPA in the free fatty acid (FFA) form has been shown to reduce adenomatous polyp number and size in patients with familial adenomatous polyposis [66]. Metabolism of omega-6 PUFAs generally results in proinflammatory mediators, whereas byproducts of omega-3 (n-3) PUFAs generally are less inflammatory. Statistically significant increases in colon cancer risk for low docosahexaenoic acid (DHA) scores in combination with high inflammatory scores (low EPA intake, high AA intake, high BMI, and smoking) have been observed, providing evidence for an interaction between dietary animal fat intake and genetic variation involved in eicosanoid metabolism and colorectal cancer risk [67]. Experimental models consistently show a modulation of carcinogenesis by omega-3 PUFAs. For example, in lung cancer, the anticancer activity of fish oils against human lung cancer is associated with changes in formation of PGE (2), PGE (3) and alteration of Akt phosphorylation [68]. 

 In breast cancer, higher intake of omega-3 fatty acids has been linked to decreased inflammation and decreased fatigue in breast cancer survivors [69]. The omega-3 long chain polyunsaturated fatty acids, docosahexaenoic acid (DHA) and EPA, elicit antiproliferative effects in cancer cell lines and in animal models. Dietary DHA and EPA can be converted to their ethanolamine derivatives, which exert antiproliferative effects by inducing autophagy in breast cancer cells. These properties could be of use as breast cancer preventive and/or therapeutic agents [70]. Also, DHA given during chemotherapy did not produce adverse side effects and improved the outcome of chemotherapy. DHA also has a potential to specifically chemosensitize tumors [71]. A study of alphalinolenic acid showed that this supplement alone might have growth inhibitory and proapoptotic effects on estrogen positive breast cancer cells [72]. In one study, breast carcinoma cell proliferation was reduced by 60% in lesions from the high n-3:n-6 treatment group compared with the low n-3:n-6 treatment group. The apoptotic index was increased in the high n-3:n-6 group. Changes in protein expression were consistent with reduced inflammation and suppressed mTOR activity, and the high n-3:n-6 treatment showed changes in PPAR*γ* activation and suppression of lipid synthesis [73].

 Exogenous PUFAs have been shown to attenuate breast cancer cell proliferation and migration, suggesting a mechanism in which PUFAs restrain breast cancer growth partly via their inhibition of transient receptor potential channels (TRPC) [74]. DHA has shown anticancer action *in vitro* and *in vivo* in a variety of cancers. A group investigated the role for DHA in inducing apoptosis in triple-negative breast cancer (TNBC) and studied the mechanisms of action. The results showed that DHA induces apoptosis in TNBC cells via activation of caspase-8 and -9 dependent proapoptotic events [75]. Expression of colony stimulating factor-1 (CSF-1) by breast cancer cells acts as potent activator of malignancy and metastasis. One study revealed a mechanism for the function of a *ω*-3 PUFA diet that blocks microRNA-21, thereby increasing the tumor suppressor gene PTEN levels which may help prevent expression of CSF-1 in breast cancer [76]. A study of 633 breast cancer survivors participating in the *Health, Eating, Activity, and Lifestyle Study* had subjects complete a food frequency/dietary supplement questionnaire and provide blood samples to assess diet and inflammatory markers [69]. The results showed that breast cancer survivors with a higher intake of *ω*-6 relative to *ω*-3 PUFAs were associated with greater C-reactive protein levels and greater odds of having fatigue (OR of 2.6 for the highest versus the lowest tertile of intake). The results suggest that reducing *ω*-6 while increasing *ω*-3 fatty acid intake might be beneficial for reducing fatigue in patients with cancer. 

 Some studies suggest that dietary intake of EPA and DHA in foods and supplements may have protective associations against the development of endometrial cancer [77]. EPA and DHA were found to induce reactive oxygen species accumulation and caspase-8-dependent cell death in human pancreatic cancer cells. In an *in vivo* study, a diet with high levels of EPA and DHA strongly suppressed the growth of human pancreatic cancer xenografts in athymic nude mice, by inducing oxidative stress and cell death [78]. In addition, *ω*-3 PUFAs have been shown to preserve muscle mass and function in cancer patients even during active treatment. During chemotherapy, omega-3 fatty acids may contribute to a reduced inflammatory response, and some small studies showed that omega-3 fatty acids increase the response rate to chemotherapy [79].

 The relationship between specific fatty acids and prostate cancer survival remains a field with need for more research. Dietary intake of 14 fatty acids was analyzed in a population-based cohort of 525 Swedish men with prostate cancer. Among all men, those with the highest omega-3 DHA and total marine fatty acid intakes were 40% less likely to die from prostate cancer.

 Complications of treatment such as neuropathy may potentially be helped by *ω*-3 PUFAs. Axonal sensory peripheral neuropathy is a major concern in paclitaxel therapy. Omega-3 fatty acids have beneficial effects on neural damage from their effects on neuronal cells and participate in inhibition of the formation of proinflammatory cytokines involved in peripheral neuropathy. Patients with breast cancer have a longer disease free survival rate with the newer therapeutic agents. Finding a way to mitigate the disabling effects of iatrogenic peripheral neuropathy may significantly improve the patients' quality of life [80]. In more complicated clinical scenarios, *ω*-3 PUFAs supplemented parenteral nutrition can reduce inflammation and improve immune function in patients following esophageal cancer surgery [81]. In view of this literature, it would seem that the use of *ω*-3 PUFAs in patients with cancer is a desirable practice. 

### 5.3. Vitamin D

In the past few years, there has been a growing emphasis on the importance of maintaining adequate levels of vitamin D to help maintain overall health and immune function. Similarly, maintaining adequate levels of vitamin D in advanced cancer and palliative care patients would seem an easy and logical strategy. The data below strongly supports the importance of achieving adequate blood levels of vitamin D in such patients. 

 From a physiological perspective, several studies support the importance of vitamin D to help with cancer management. The active form of vitamin D interacts with the vitamin D receptor (VDR) to induce antiproliferative, anti-invasive, proapoptotic, and prodifferentiation activities in prostate cancer cells [82]. Via several different hydroxylase enzymes, prostate tissue appears to have the ability to activate and inactivate vitamin D in an autocrine/paracrine fashion. Recent evidence indicates that 25-hydroxyvitamin D [25(OH)D] can bind to the VDR to modulate gene expression that can lead to the arrest of cell growth. In fact, preclinical data have indicated that vitamin D affects up to 200 genes that influence cellular proliferation, apoptosis, angiogenesis, terminal differentiation of normal and cancer cells, and macrophage function [83]. Thus, circulating levels of 25(OH)D may play an important role in the body's ability to regulate the growth of prostate cancer. Similarly, while the evidence regarding the association of vitamin D levels and hepatocellular carcinoma (HCC) and pancreatic cancer is inconclusive at the present time, biochemical evidence suggests that both HCC and pancreatic cancer cells are responsive to the inhibitory effects of vitamin D [84, 85].

 Clinical studies have also suggested an important role for vitamin D in cancer. To begin, several studies have shown that patients with cancer often have low levels of vitamin D. For example, a study of 195 patients presenting to a radiation oncology clinic with advanced stages of cancer showed that approximately 75% had vitamin D levels that were either deficient (<20 ng/mL) or suboptimal (20–30 ng/mL) [86]. The authors concluded that low serum vitamin D levels, independent of age, sex, and body mass index, predicted advanced stage disease. An evaluation of 391 postmenopausal women with stage I–III breast cancer on aromatase inhibitor therapy found that 35% of the women were vitamin D deficient [87]. Overall, vitamin D insufficiency may be present in up to 75% of women with breast cancer [83].

 Studies have reported an inverse relationship between vitamin D intake and the risk of breast cancer. Studies have also suggested that there are improvements in cancer survival after a diagnosis of breast cancer in women when they have higher levels of vitamin D [83]. Vitamin D receptors have been found in up to 80% of breast cancers, and VDR polymorphisms have been associated with differences in survival. Although ongoing studies have investigated a possible link between adequate levels of vitamin D and improved cancer prognosis, breast cancer survivors may derive additional, non-cancer-related benefits from adequate vitamin D levels, including improvements in bone mineral density, quality of life, and mood. 

 Another study of 658 patients with various types of cancer showed that patients with 25(OH)D levels below 46 nmol/L at diagnosis experienced shorter survival [88]. In addition, those patients in the highest quartile had a significantly reduced risk of death compared to the lowest quartile. A study by Cheng and Neuhouser [89] analyzed 16,693 men and women in the Third National Health and Nutrition Examination Survey (NHANES III) between 1988 and 1994 to determine the relationship between vitamin D levels and lung cancer mortality. The results showed that among nonsmokers, a vitamin D level ≥44 versus <44 nmol/L was associated with a decreased risk of developing lung cancer (HR = 0.53 in former/never smokers and HR = 0.31 in distant-former (quit ≥ 20 years)/never smokers). Interestingly, this relationship was not found in smokers. Furthermore, the beneficial association was diminished among those patients with excess circulating vitamin A or vitamin A/*β*-carotene supplement users.

 In patients with ovarian cancer, serum concentration of 25(OH)D_3_ was lower than that found in a noncancer reference group [90]. In addition, the 5-year survival rate was significantly higher in the subgroup of patients with 25(OH)D_3_ concentrations over 10 ng/mL (46% survival) compared to women with concentrations below 10 ng/mL (26% survival). Another study of 1800 patients with breast cancer showed that serum 25OHD (>30 ng/mL) at initial diagnosis of breast cancer significantly correlated with smaller tumor size, improved overall survival, and disease specific survival, especially in postmenopausal patients [91].

 Of course, while the association between low vitamin D levels and cancer is supported by a number of the mentioned studies, perhaps the more important question is whether vitamin D supplementation will actually be beneficial in the management of cancer patients, and for this paper, whether such treatment might be useful and advisable in the palliative care population. One study showed that when 69 vitamin D deficient patients were given 2,000 u vitamin D per day, there was a marked improvement from baseline in fatigue (*P* < 0.05) after 3 months which also corresponded to improved serum levels of vitamin D [92]. Importantly, the authors report that the safety profile of vitamin D in combination with chemotherapy was acceptable. 

 In patients with low-risk prostate cancer, in those receiving 4,000 IU/d for one year [93], no adverse events were associated with vitamin D_3_ supplementation. The results demonstrated that 55% of patients had a decrease in the number of positive cores or decrease in Gleason score; 11% showed no change; and 34% showed an increase in the number of positive cores or Gleason score. Since this was not a randomized study, it is difficult to make any firm conclusions, but the data suggests a potential benefit from vitamin D supplementation in these patients.

 A double-blind placebo-controlled randomized trial of high dose vitamin D supplementation (50,000 IU per week) in 60 women with breast cancer and aromatase inhibitor-induced musculoskeletal symptoms showed that, at 2 and 6 months, measures of pain such as the FIQ pain, BPI worst-pain, BPI average-pain, BPI pain-severity, and BPI pain interference scores were significantly better in those patients receiving vitamin D compared to placebo [94]. 

 Few studies have actually been performed in palliative care patients, but given the above data, it would seem reasonable to ensure that serum vitamin D levels are maintained. Vitamin D supplementation would likely be beneficial both with regard to the cancer itself and perhaps more importantly for the palliative care patient, helping to maintain quality of life. In addition, there appears to be a very little problem with providing vitamin D supplementation in terms of adverse effects or interference with medications. Thus, adequate vitamin D supplementation to maintain a serum level of at least 50 ng/mL would seem to be an appropriate nutritional goal in palliative care patients. This could be done with either daily supplementation or weekly typically with doses of vitamin D_3_ between 3,000 and 5,000 IU daily which generally achieves adequate levels in most treated patients with a target of approximately 50 ng/mL. Consideration should also be given to include supplementation of vitamin D cofactors such as calcium and phosphate in the appropriate setting, especially when deficient.

### 5.4. Multivitamins and Antioxidants

Even multivitamins could potentially have an emerging role in the management of cancer patients in general and in palliative care patients specifically. Kwan et al. [95] evaluated vitamin supplement use and exercise in 2,236 women diagnosed with early stage breast cancer and found that women who consistently used multivitamins before and after diagnosis, ate more fruits/vegetables, and were more physically active had better overall survival. However, it is interesting to note that those patients who began vitamin supplement use after the diagnosis of breast cancer did not demonstrate any advantage.

 Another controversy regards the use of antioxidant supplements during chemotherapy treatment. Although traditionally this has not been considered good practice, there is mounting evidence that antioxidants may be helpful. A systematic analysis of 19 randomized, controlled clinical trials of antioxidant concurrent use in chemotherapy patients revealed that in most studies, antioxidant supplementation resulted in either increased survival times, increased tumor responses, or both, as well as fewer toxicities than controls [96]. The supplements evaluated in these studies included glutathione, melatonin, vitamin A, an antioxidant mixture, vitamin C, N-acetylcysteine, vitamin E, and ellagic acid. The report noted that no studies indicated a poorer outcome or an interference between the antioxidant and chemotherapy. However, the authors noted that the studies generally suffered from a lack of adequate statistical power and were too heterogeneous in their design to draw any strong conclusions.

 Another controversial area is antioxidant supplements during radiation. Many acute and chronic effects of ionizing radiation are mediated by reactive oxygen species and reactive nitrogen species which deplete antioxidant stores, leading to cellular apoptosis, stem cell depletion, and accelerated aging. Epperly et al. [97] have found that survivors of acute ionizing radiation damage have ameliorated life shortening if they are fed an antioxidant-chemopreventive diet.

 In recent years, a large number of studies have attributed a protective effect to polyphenols and foods containing these compounds (e.g., cereals, coffee, dark chocolate, plants, tea, or vegetables) against cancer development and propagation, when used as chemopreventive agents. The mechanism of action of these polyphenols is most likely based upon antioxidant activity [98]. However, studies have also suggested that these compounds may inhibit cancer growth by exerting prooxidant effects or affecting the growth factor-mediated pathway, the mitogen-activated protein kinase-dependent pathway, and the ubiquitin/proteasome degradation pathways [99]. Some polyphenols reported a preventive action against colon cancer, for example, curcumin, gallic acid, ellagic acid, and epigallocatechin-3-gallate [100].

## 6. Conclusions

Developing a healthier diet with the addition of specific supplements designed to reduce oxidative stress and inflammation should be an important part of an overall palliative care plan. It is interesting to note that the cancer diagnosis is frequently a motivator for improving diet and nutrition. A study of 1,560 patients with breast cancer [1] found that intake of fruit and vegetables, whole grains, and lean sources of protein increased significantly after diagnosis, while consumption of high-fat, high-sugar products, red meat, coffee, some alcoholic drinks, and refined grains significantly decreased. The postdiagnostic changes in diet were accompanied by changes in the intake of macronutrients and a number of vitamins and minerals. Supplement use such as fish oils, multivitamin and minerals, and evening primrose oil was increased after diagnosis. The importance of this study is that people can truly alter their dietary and nutritional habits when enough motivating factors are present. This is encouraging data that supports the importance of modifying diets for palliative care patients as well. Sometimes, patients and their families also feel more positively, with a stronger sense of control over their circumstances, when they know they are engaged in behaviors that could benefit their overall health outcome. We strongly believe that physicians should be encouraged to find occasions to impart appropriate nutritional, physical activity, and weight management guidance to their patients whenever possible. The appropriate teaching, offered with physician authority combined with an attitude of caring, when given at the right time, can have a life changing effect for a patient. 

 The majority of cancer fighting nutrients should be obtained via a wholesome diet. Nutrient-dense foods contain substantial amounts of key nutrients in relation to the dietary energy they provide [101]. Nutrient dense calories include fruits, vegetables, nuts, and select high quality dairy products and meats [102]. Furthermore, vegetarian diets are associated with lower risk for cancer [103]. The evidence is mounting that diets based upon these foods can be beneficial to patients with cancer in survivorship and possibly for prevention of metastases or recurrences. In addition, a nutrient-dense food diet should be combined with choosing food for freshness, wholesomeness, and a decrease in the degree of processing. Importantly, food should also be, as much as possible, a pleasurable experience since a positive food experience can help provide comfort and a sense of normality for palliative care patients.

 In conclusion, it is important to consider the diet and nutritional planning for patients in the palliative care setting, focusing on foods and supplements that provide cancer fighting nutrients, reduce oxidative stress and inflammation can help overall quality of life, and have a beneficial effect on their cancer and other medical conditions. Anything done by the patients on their own to take an active role in improving health and cancer outcomes is desirable on multiple levels. This is an era of increasing current and future global cancer burden, coupled with concomitantly shrinking health care budgets and resources. Hopefully, this paper serves as stimulus for further research and activity in exploring the concept of dietary intervention in palliative care patients with cancer as a possible treatment plan costrategy for changing the “host” in ways which favor physiological function of the whole body and contemporaneously oppose cancer biology, while addressing caloric, macro, and micronutritional requirements.

With the projected global future cancer burden and attendant health care costs, every opportunity should be taken to teach patients and, therefore, their close contacts behaviors which could positively influence the health of the population at large.

## References

[1] Velentzis LS, Keshtgar MR, Woodside JV (2011). Significant changes in dietary intake and supplement use after breast cancer diagnosis in a UK multicentre study. *Breast Cancer Research and Treatment*.

[2] Nixon DW, Kutner M, Heymsfield S (1988). Resting energy expenditure in lung and colon cancer. *Metabolism*.

[3] Falconer JS, Fearon KCH, Plester CE, Ross JA, Carter DC (1994). Cytokines, the acute-phase response, and resting energy expenditure in cachectic patients with pancreatic cancer. *Annals of Surgery*.

[4] Dempsey DT, Feurer ID, Knox LS (1984). Energy expenditure in malnourished gastrointestinal cancer patients. *Cancer*.

[5] Doyle C, Kushi LH, Byers T (2006). Nutrition and physical activity during and after cancer treatment: an American cancer society guide for informed choices. *CA Cancer Journal for Clinicians*.

[6] Wallengren O, Bosaeus I, Lundholm K (2012). Dietary energy density is associated with energy intake in palliative care cancer patients. *Supportive Care in Cancer*.

[7] Bruera E, Strasser F, Palmer JL (2003). Effect of fish oil on appetite and other symptoms in patients with advanced cancer and anorexia/cachexia: a double-blind, placebo-controlled study. *Journal of Clinical Oncology*.

[8] Winkler M (2004). Body compositional changes in cancer cachexia: are they reversible?. *Topics in Clinical Nutrition*.

[9] Deans C, Wigmore SJ (2005). Systemic inflammation, cachexia and prognosis in patients with cancer. *Current Opinion in Clinical Nutrition and Metabolic Care*.

[10] Rockenbach G, di Pietro PF, Ambrosi C (2011). Dietary intake and oxidative stress in breast cancer: before and after treatments. *Nutrición Hospitalaria*.

[11] Kwan ML, Weltzien E, Kushi LH, Castillo A, Slattery ML, Caan BJ (2009). Dietary patterns and breast cancer recurrence and survival among women with early-stage breast cancer. *Journal of Clinical Oncology*.

[12] Villarini A, Pasanisi p, Raimondi M (2012). Preventing weight gain during adjuvant chemotherapy for breast cancer: a dietary intervention study. *Breast Cancer Research and Treatment*.

[13] Demark-Wahnefried W, Campbell KL, Hayes SC (2012). Weight management and its role in breast cancer rehabilitation. *Cancer*.

[14] Mosby TT, Cosgrove M, Sarkardei S S, Platt KL, Kaina B (2012). Nutrition in adult and childhood cancer: role of carcinogens and anti-carcinogens. *Anticancer Research*.

[15] Drake I, Sonestedt E, Gullberg B (2012). Dietary intakes of carbohydrates in relation to prostate cancer risk: a prospective study in the Malmö diet and cancer cohort. *American Journal of Clinical Nutrition*.

[16] Liu H, Heaney AP (2011). Refined fructose and cancer. *Expert Opinion on Therapeutic Targets*.

[17] Port AM, Ruth MR, Istfan NW (2012). Fructose consumption and cancer: is there a connection?. *Current Opinion in Endocrinology Diabetes and Obesity*.

[18] Cohen DH, LeRoith D (2012). Obesity, type 2 diabetes, and cancer: the insulin and IGF connection. *Endocrine Related Cancer*.

[19] Gallagher EJ, LeRoith D (2010). The proliferating role of insulin and insulin-like growth factors in cancer. *Trends in Endocrinology and Metabolism*.

[20] Hebels DGAJ, Sveje KM, de Kok MC (2012). Red meat intake-induced increases in fecal water genotoxicity correlate with pro-carcinogenic gene expression changes in the human colon. *Food and Chemical Toxicology*.

[21] Tang F-Y, Pai M-H, Chiang E-PI (2012). Consumption of high-fat diet induces tumor progression and epithelial-mesenchymal transition of colorectal cancer in a mouse xenograft model. *Journal of Nutritional Biochemistry*.

[22] Corpet DE (2011). Red meat and colon cancer: should we become vegetarians, or can we make meat safer?. *Meat Science*.

[23] Meyerhardt JA (2011). Beyond standard adjuvant therapy for colon cancer: role of nonstandard interventions. *Seminars in Oncology*.

[24] Montonen J, Boeing H H, Fritsche A (2012). Consumption of red meat and whole-grain bread in relation to biomarkers of obesity, inflammation, glucose metabolism and oxidative stress. *European Journal of Nutrition*.

[25] Wu JW, Cross AJ, Baris D (2012). Dietary intake of meat, fruits, vegetables, and selective micronutrients and risk of bladder cancer in the New England region of the United States. *British Journal of Cancer*.

[26] Sethi G, Shanmugam MK, Ramachandran L, Kumar AP, Tergaonkar V (2012). Multifaceted link between cancer and inflammation. *Bioscience Reports*.

[27] Nathan C, Cunningham-Bussel A (2013). Beyond oxidative stress: an immunologist's guide to reactive oxygen species. *Nature Reviews in Immunology*.

[28] Sumantran VN, Tillu G (2012). Cancer, inflammation, and insights from ayurveda. *Evidence Based Complementary and Alternative Medicine*.

[29] McCarty MF (2011). mTORC1 activity as a determinant of cancer risk—rationalizing the cancer-preventive effects of adiponectin, metformin, rapamycin, and low-protein vegan diets. *Medical Hypotheses*.

[30] Fernández MA, De Las Heras B, García MD, Sáenz MT, Villar A (2001). New insights into the mechanism of action of the anti-inflammatory triterpene lupeol. *Journal of Pharmacy and Pharmacology*.

[31] Yadav VR, Prasad S, Sung B, Kannappan R, Aggarwal BB (2010). Targeting inflammatory pathways by triterpenoids for prevention and treatment of cancer. *Toxins*.

[32] Gingras D, Béliveau R (2011). Colorectal cancer prevention through dietary and lifestyle modifications. *Cancer Microenvironment*.

[33] Tantamango-Bartley Y, Jaceldo-Siegl K, Fan J, Fraser G (2013). Vegetarian diets and the incidence of cancer in a low-risk population. *Cancer Epidemiology, Biomarkers & Prevention*.

[34] Haydé VC, Ramón GG, Lorenzo GO (2012). Non-digestible fraction of beans (*Phaseolus vulgaris*L.) modulates signalling pathway genes at an early stage of colon cancer in Sprague-Dawley rats. *British Journal of Nutrition*.

[35] Pietro Femia A, Luceri C, Bianchini F (2012). Marie Ménard apples with high polyphenol content and a low-fat diet reduce 1, 2-dimethylhydrazine-induced colon carcinogenesis in rats: effects on inflammation and apoptosis. *Molecular Nutrition Food Research*.

[36] Knackstedt RW, Moseley VR, Wargovich MJ (2012). Epigenetic mechanisms underlying diet-sourced compounds in the prevention and treatment of gastrointestinal cancer. *Anticancer Agents in Medicinal Chemistry*.

[37] Dougherty U, Mustafi R, Wang Y (2011). American ginseng suppresses Western diet-promoted tumorigenesis in model of inflammation-associated colon cancer: role of EGFR. *BMC Complementary and Alternative Medicine*.

[38] Monti DA, Bazzan AJ (2008). *The Great Life Makeover*.

[39] Abd El-Atti S, Wasicek K, Mark S, Hegazi R (2009). Use of probiotics in the management of chemotherapy-induced diarrhea: a case study. *Journal of Parenteral and Enteral Nutrition*.

[40] Boleij A, Tjalsma H (2012). Gut bacteria in health and disease: a survey on the interface between intestinal microbiology and colorectal cancer. *Biological Reviews*.

[41] de Moreno de LeBlanc A, Matar C, Perdigón G (2007). The application of probiotics in cancer. *British Journal of Nutrition*.

[42] Wanchai A, Armer JM, Stewart BR (2010). Complementary and alternative medicine use among women with breast cancer: a systematic review. *Clinical Journal of Oncology Nursing*.

[43] Astin JA, Reilly C, Perkins C, Child WL (2006). Breast cancer patients’ perspectives on and use of complementary and alternative medicine: a study by the Susan G. Komen Breast Cancer Foundation. *Journal of the Society for Integrative Oncology*.

[44] Jirillo E, Jirillo F, Magrone T (2012). Healthy effects exerted by prebiotics, probiotics, and symbiotics with special reference to their impact on the immune system. *International Journal of Vitamin Nutrition Research*.

[45] Patel RM, Denning PW (2013). Therapeutic use of prebiotics, probiotics, and postbiotics to prevent necrotizing enterocolitis: what is the current evidence?. *Clinical Perinatology*.

[46] Gallimore AM, Godkin A (2013). Epithelial barriers, microbiota, and colorectal cancer. *The New England Journal of Medicine*.

[47] Kumar M, Nagpal R, Verma V (2013). Probiotic metabolites as epigenetic targets in the prevention of colon cancer. *Nutrition Reviews*.

[48] Verma A, Shukla G (2013). Probiotics Lactobacillus rhamnosus GG, Probiotics *Lactobacillus rhamnosus* GG, *Lactobacillus acidophilus* suppresses DMH-induced procarcinogenic fecal enzymes and preneoplastic aberrant crypt foci in early colon carcinogenesis in Sprague Dawley rats. *Nutrition and Cancer*.

[49] Di Bartolomeo F, Startek JB, Van den Ende W (2012). Prebiotics to fight diseases: reality or fiction?. *Phytotherapy Research*.

[50] Otieno DO, Ahring BK BK (2012). The potential for oligosaccharide production from the hemicellulose fraction of biomasses through pretreatment processes: xylooligosaccharides (XOS), arabinooligosaccharides (AOS), and mannooligosaccharides (MOS). *Carbohydrate Research*.

[51] Actis GC, Tarallo S, Rosina F (2012). Cutting edge: chemoprevention of colorectal neoplasia in inflammatory bowel disease.. *Inflammation and Allergy Drug Targets*.

[52] Bassaganya-Riera J, Viladomiu M, Pedragosa M, De Simone C, Hontecillas R (2012). Immunoregulatory mechanisms underlying prevention of colitis-associated colorectal cancer by probiotic bacteria. *PLoS One*.

[53] Orlando A, Russo F (2013). Intestinal microbiota, probiotics and human gastrointestinal cancers. *Journal of Gastrointestinal Cancer*.

[54] Uccello M, Malaguarnera G, Basile F (2012). Potential role of probiotics on colorectal cancer prevention. *BMC Surgery*.

[55] Visich KL, Yeo TP (2010). The prophylactic use of probiotics in the prevention of radiation therapy-induced diarrhea. *Clinical Journal of Oncology Nursing*.

[56] Delia P, Sansotta G, Donato V (2007). Use of probiotics for prevention of radiation-induced diarrhea. *World Journal of Gastroenterology*.

[57] Delia P, Sansotta G, Donato V (2002). Prophylaxis of diarrhoea in patients submitted to radiotherapeutic treatment on pelvic district: personal experience. *Digestive and Liver Disease*.

[58] Bowen JM, Stringer AM, Gibson RJ, Yeoh ASJ, Hannam S, Keefe DMK (2007). VSL#3 probiotic treatment reduces chemotherapy-induced diarrhea and weight loss. *Cancer Biology and Therapy*.

[59] Österlund P, Ruotsalainen T, Korpela R (2007). Lactobacillus supplementation for diarrhoea related to chemotherapy of colorectal cancer: a randomised study. *British Journal of Cancer*.

[60] Aagaard K, Petrosino J, Keitel W (2012). The human microbiome project strategy for comprehensive sampling of the human microbiome and why it matters. *FASEB Jounal*.

[61] Devaraj S, Hemarajata P, Versalovic J (2013). The human gut microbiome and body metabolism: implications for obesity and diabetes. *Clinical Chemistry*.

[62] Peitsidou K, Karantanos T, Theodoropoulos GE (2012). Probiotics, prebiotics, synbiotics: is there enough evidence to support their use in colorectal cancer surgery?. *Digestive Surgery*.

[63] Johnston BC, Ma SS, Goldenberg JZ (2012). Probiotics for the prevention of clostridium difficile-associated diarrhea: a systematic review and meta-analysis. *Annals of Internal Medicine*.

[64] Boyle RJ, Robins-Browne RM, Tang MLK (2006). Probiotic use in clinical practice: what are the risks?. *American Journal of Clinical Nutrition*.

[65] Vanden Heuvel JP (2012). Nutrigenomics and nutrigenetics of *ω*3 polyunsaturated fatty acids. *Progress in Molecular Biology and Translational Science*.

[66] Hull MA (2013). Nutritional agents with anti-inflammatory properties in chemoprevention of colorectal neoplasia. *Recent Results in Cancer Research*.

[67] Habermann N, Ulrich CM, Lundgreen A (2013). PTGS1, PTGS2, ALOX5, ALOX12, ALOX15, and FLAP SNPs: interaction with fatty acids in colon cancer and rectal cancer. *Genes and Nutrition*.

[68] Yang P, Cartwright C, Chan D (2013). Anticancer activity of fish oils against human lung cancer is associated with changes in formation of PGE(2) and PGE(3) and Alteration of Akt Phosphorylation. *Molecular Carcinogenesis*.

[69] Alfano CM, Imayama I, Neuhouser ML (2012). Fatigue, inflammation, and *ω*-3 and *ω*-6 fatty acid intake among breast cancer survivors. *Journal of Clinical Oncology*.

[70] Rovito D, Giordano C, Vizza D (2012). Omega-3 PUFA ethanolamines DHEA and EPEA induce autophagy through PPAR*γ* activation in MCF-7 breast cancer cells. *Journal of Cellular Physiology*.

[71] Bougnoux P, Hajjaji N, Maheo K, Couet C, Chevalier S (2010). Fatty acids and breast cancer: sensitization to treatments and prevention of metastatic re-growth. *Progress in Lipid Research*.

[72] Kim J-Y, Park HD, Park E, Chon J-W, Park YK (2009). Growth-inhibitory and proapoptotic effects of alpha-linolenic acid on estrogen-positive breast cancer cells: second look at n-3 fatty acid. *Annals of the New York Academy of Sciences*.

[73] Jiang W, Zhu Z, McGinley JN, El Bayoumy K, Manni A, Thompson HJ (2012). Identification of a molecular signature underlying inhibition of mammary carcinoma growth by dietary N-3 fatty acids. *Cancer Research*.

[74] Zhang H, Zhou L, Shi W, Song N, Yu K, Gu Y (2012). A mechanism underlying the effects of polyunsaturated fatty acids on breast cancer. *International Journal of Molecular Medicine*.

[75] Xiong A, Yu W, Tiwary R, Sanders BG, Kline K (2012). Distinct roles of different forms of vitamin E in DHA-induced apoptosis in triple-negative breast cancer cells. *Molecular Nutrition and Food Research*.

[76] Mandal CC, Ghosh-Choudhury T, Dey N, Choudhury GG, Ghosh-Choudhury N (2012). miR-21 is targeted by omega-3 polyunsaturated fatty acid to regulate breast tumor CSF-1 expression. *Carcinogenesis*.

[77] Arem H, Neuhouser ML, Irwin ML ML (2012). Omega-3 and omega-6 fatty acid intakes and endometrial cancer risk in a population-based case-control study. *European Journal of Nutrition*.

[78] Fukui M, Kang KS, Okada K, Zhu BT BT (2013). EPA, an omega-3 fatty acid, induces apoptosis in human pancreatic cancer cells: role of ROS accumulation, caspase-8 activation, and autophagy induction. *Journal of Cellular Biochemistry*.

[79] Epstein MM, Kasperzyk JL, Mucci LA (2012). Dietary fatty acid intake and prostate cancer survival in Örebro County, Sweden. *American Journal of Epidemiology*.

[80] Ghoreishi Z, Esfahani A, Djazayeri A (2012). Omega-3 fatty acids are protective against paclitaxel-induced peripheral neuropathy: a randomized double-blind placebo controlled trial. *BMC Cancer*.

[81] Long H, Yang H, Lin Y, Situ D, Liu W (2013). Fish oil-supplemented parenteral nutrition in patients following esophageal cancer surgery: effect on inflammation and immune function. *Nutrition and Cancer*.

[82] Chen TC, Sakaki T, Yamamoto K, Kittaka A (2012). The roles of cytochrome P450 enzymes in prostate cancer development and treatment. *Anticancer Research*.

[83] Hines SL, Jorn HKS, Thompson KM, Larson JM (2010). Breast cancer survivors and vitamin D: a review. *Nutrition*.

[84] Chiang K-C, Yeh C-N, Chen M-F, Chen TC (2011). Hepatocellular carcinoma and vitamin D: a review. *Journal of Gastroenterology and Hepatology*.

[85] Chiang K-C, Yeh C-N, Chen TC (2011). Vitamin D and pancreatic cancer-an update. *Cancers*.

[86] Churilla TM, Brereton HD, Klem M, Peters CA (2012). Vitamin D deficiency is widespread in cancer patients and correlates with advanced stage disease: a community oncology experience. *Nutrition Cancer*.

[87] Friedman CF, DeMichele A, Su HI (2012). Vitamin D deficiency in postmenopausal breast cancer survivors. *Journal of Women’s Health*.

[88] Tretli S, Schwartz GG, Torjesen PA, Robsahm TE (2012). Serum levels of 25-hydroxyvitamin D and survival in Norwegian patients with cancer of breast, colon, lung, and lymphoma: a population-based study. *Cancer Causes and Control*.

[89] Cheng TY, Neuhouser ML (2012). Serum 25-hydroxyvitamin D, vitamin A, and lung cancer mortality in the US population: a potential nutrient-nutrient interaction. *Cancer Causes and Control*.

[90] Walentowicz-Sadlecka M, Grabiec M, Sadlecki P P (2012). 25(OH)D3 in patients with ovarian cancer and its correlation with survival. *Clinical Biochemistry*.

[91] Hatse S, Lambrechts D, Verstuyf A (2012). Vitamin D status at breast cancer diagnosis: correlation with tumor characteristics, disease outcome, and genetic determinants of vitamin D insufficiency. *Carcinogenesis*.

[92] Trivanovic D, Plestina S, Dobrilla-Dintinjana R (2012). Vitamin D3 supplementation to improve fatigue in patients with advanced cancer. *Journal of Clinical Oncology*.

[93] Marshall DT, Savage SJ, Garrett-Mayer E E (2012). Vitamin D3 supplementation at 4000 international units per day for one year results in a decrease of positive cores at repeat biopsy in subjects with low-risk prostate cancer under active surveillance. *The Journal of Clinical Endocrinology & Metabolism*.

[94] Rastelli AL, Taylor ME, Gao F (2011). Vitamin D and aromatase inhibitor-induced musculoskeletal symptoms (AIMSS): a phase II, double-blind, placebo-controlled, randomized trial. *Breast Cancer Research and Treatment*.

[95] Kwan ML, Greenlee H, Lee VS (2011). Multivitamin use and breast cancer outcomes in women with early-stage breast cancer: the life after cancer epidemiology study. *Breast Cancer Research and Treatment*.

[96] Block KI, Koch AC, Mead MN, Tothy PK, Newman RA, Gyllenhaal C (2007). Impact of antioxidant supplementation on chemotherapeutic efficacy: a systematic review of the evidence from randomized controlled trials. *Cancer Treatment Reviews*.

[97] Epperly MW, Wang H, Jones JA, Dixon T, Montesinos CA, Greenberger JS (2011). Antioxidant-chemoprevention diet ameliorates late effects of total-body irradiation and supplements radioprotection by MnSOD-plasmid liposome administration. *Radiation Research*.

[98] Forester SC, Lambert JD (2011). The role of antioxidant versus pro-oxidant effects of green tea polyphenols in cancer prevention. *Molecular Nutrition and Food Research*.

[99] Chen D, Milacic V, Chen MS (2008). Tea polyphenols, their biological effects and potential molecular targets. *Histology and Histopathology*.

[100] Santos IS, Ponte BM, Boonme P, Silva AM, Souto EB (2012). Nanoencapsulation of polyphenols for protective effect against colon-rectal cancer. *Biotechnology Advances*.

[101] Darmon N, Darmon M, Maillot M, Drewnowski A (2005). A nutrient density standard for vegetables and fruits: nutrients per calorie and nutrients per unit cost. *Journal of the American Dietetic Association*.

[102] McEvoy CT, Temple N, Woodside JV (2012). Vegetarian diets, low-meat diets and health: a review. *Public Health and Nutrition*.

[103] Huang T, Yang B, Zheng J, Li G, Wahlqvist ML, Li D (2012). Cardiovascular disease mortality and cancer incidence in vegetarians: a meta-analysis and systematic review. *Annals of Nutrition and Metabolism*.

